# Management of Failed Surgery for Anterior Glenohumeral Instability: Synopsis of Clinical Evidence

**DOI:** 10.5041/RMMJ.10408

**Published:** 2020-10-14

**Authors:** Nahum Rosenberg, Kamal Hamoud

**Affiliations:** 1 The Ruth & Bruce Rappaport Faculty of Medicine, Technion–Israel Institute of Technology, Haifa, Israel; 2 Muscle and Skeleton Laboratory, Orthopedic Surgery Section, Rambam Health Care Campus, Haifa, Israel; 3 Back Unit, Orthopedic Surgery Section, Rambam Health Care Campus, Haifa, Israel

**Keywords:** Engaging Hill-Sachs, glenoid deficiency, remplissage, shoulder instability, shoulder stabilization

## Abstract

Failed surgical treatment of anterior shoulder instability should be treated according to clinical principles similar to primary stabilization by addressing risk factors related to the damaged static glenohumeral stabilizers (labrum, capsule and its components, and bony damage to the humeral head and scapular glenoid). In relatively rare conditions when failed primary surgery involves patients with functionally low demands, conservative treatment by strengthening dynamic muscular stabilizers might be considered; otherwise, surgical revision should be strongly considered aimed at improving quality of life. Although the overall failure rate following primary and revision surgery is expected to be below 4%, it is clear that revision surgery is technically demanding. Therefore, the initial recognition and correction of the exact pathology causing glenohumeral instability is crucial to avoid failure of primary surgery and to facilitate the success of the revision procedure, if necessary.

## BACKGROUND

Primary surgery for anterior shoulder stabilization fails in up to 30% of patients, but the reported failure rate can be as low as 3%.^1^–^3^ Naturally, this wide range of surgical failures is based on the surgical technique, the surgeon’s experience, and the patient’s compliance, but also on the definition of surgical “failure.” Obviously, re-dislocation or persistent subluxations of the glenohumeral joint can be defined as failure of the initial surgery, but persistent shoulder pain and shoulder stiffness cannot be neglected and can usually also be defined as surgical failure.^2^,^4^

Interestingly, a similar failure rate is reported by several authors after primary and revision surgery, following failed primary shoulder stabilization, either open or arthroscopic.^1^,^3^ This might indicate similar factors for surgical failure in primary and revision surgery regardless of the more technically demanding requirements in revision surgery due to a disturbed local anatomical plane and a higher grade of damage to soft tissue and bony structures. Therefore, it is logical to assume that the success of revision surgery should rely on recognition of anterior shoulder instability risk factors, which were either not addressed in primary surgery, and/or appeared as new local damage following an inadequate healing process, or as a result of a new injury.

Accordingly, with the aim of understanding the mode and source of a failed primary surgery and predicting and avoiding recurrent surgical failure, heavy emphasis has been placed on understanding and defining the risk factors of anterior shoulder stabilization failure.

To improve decision-making in planning revision surgery, several scoring systems have been developed that take into consideration demographic and anatomic factors. The highly popularized ISIS (Instability Shoulder Index Score), which takes into consideration age, sports involvement, joint laxity, and bony damage, was validated to predict surgical outcome and accordingly determine the decision-making for the preferred surgical approach and technique.^5^ This scoring system has received some criticism, especially in terms of bony damage evaluation and subjective outcomes,^6^,^7^ but overall it serves as a useful tool for presurgical planning. For a more subjective evaluation of shoulder stabilization surgery, a questionnaire-based scoring such as WOSI (Western Ontario Shoulder Instability Index) could be used.^6^ However, this type of scoring is usually useful for follow-up in order to define the success or failure of stabilization surgery and cannot be used for defining the mode of failure or for decision-making in planning revision surgery.

## THE RISK FACTORS

Recognizing the risk factors might explain the reason for the failure of primary stabilization surgery and could help in planning revision surgery and predict the risk of its failure. The risk factors include demographic and behavioral factors, type of surgical techniques used, and extent of anatomical damage.

A consensus exists that young age is a factor that highly increases re-dislocation rate following stabilization surgery. Patients younger than 20 years have a two-fold higher risk of postsurgical shoulder dislocation.^8^,^9^ The reason for this is not clear and could be attributed to higher ligament laxity, which in itself is a risk factor for recurrent dislocation, or due to less developed dynamic stabilizing musculature. Even less clear, but an obvious risk factor for re-dislocation is male gender, regardless of involvement in collision sports, with a nine-fold higher risk of new dislocation following stabilization surgery.^8^

A highly controversial risk factor is involvement in contact sports. A patient usually returns to sports activity when the treating surgeon is convinced of sufficient recovery following stabilization surgery and the rehabilitation program. It is logical to assume that in this case the anatomical etiology for the shoulder instability has been resolved, therefore the new shoulder dislocation might be considered a new injury, independent of the previous reason for treating the shoulder instability. Thus, the decision regarding the sports activity factor should be considered cautiously, providing that the surgeon and the patient are convinced of the success of the initial surgery.

Surgical pitfalls and errors in primary surgery probably have a crucial effect on the risk of failure. Anatomical deficiencies left untreated in primary surgery is probably the most important reason for the failure. Regarding the soft tissue, insufficient balancing of the redundant anterior capsule when the stabilizing anchors are placed incorrectly on the anterior glenoid rim (too lateral, too medial, or too superior), insufficient repair avulsion of the anterior glenoid labrum, and unfixed thorn humeral side of capsule leave insufficiency of the static stabilization of the capsule unsolved and highly susceptible for re-dislocation. The number of anchors that are desirable for soft tissue stabilization has recently become a subject of debate. The dogmatic rule of the required use of at least three anchors for anterior labral stabilization, which had been previously well established,^10^ has been recently challenged by showing that one or two anchors for this purpose might be sufficient.^11^ Naturally, the latter approach is advantageous in terms of cost and length of surgery. Moreover, a large number of anchors inserted into the anterior glenoid rim can cause excessive non-physiological stress and subsequentially bony avulsion of the anterior glenoid, termed “postage-stamp fracture.”^12^ Additional studies are required to compare the two surgical methods. There are currently not enough controlled studies to resolve this issue.

One important reason for primary surgery failure is the technical errors of bone block placement that cause its malunion or non-union on the anterior glenoid rim or hardware penetration into the glenohumeral joint. When bone block placement fails, in addition to the unsolved problem of glenohumeral instability, pain and stiffness of the shoulder due to non-union and/or joint surface damage become part of the surgical failure risk factors.

Bony deficiencies, either glenoid or humeral, become an important risk factor for surgical failure when they exceed a critical size, mainly according to their three-dimensional configuration. Posterolateral humeral head impression (Hill–Sachs lesion) becomes a significant risk factor for anterior shoulder instability when it exceeds 20%–25% of the humeral head spherical surface,^3^ especially if the humeral head impression’s long axis is parallel to the anterior glenoid rim and is situated on the humeral head in a position that could cause slippage over the anterior glenoid rim during functional, not extensive, shoulder movement, usually during abduction and external rotation (engaging Hill–Sachs lesion).^2^,^13^ Isolated bony glenoid deficiency usually causes anterior shoulder instability when it exceeds 20%–30%, as estimated by computed tomography (CT) imaging by approximation of the lower glenoid to the spherical shape,^14^ or by direct anterior–posterior measurements during glenohumeral arthroscopy.^15^

The combination of engaging Hill–Sachs lesion with anterior glenoid deficiency is considered a significant risk factor for anterior shoulder instability according to the “off-track” concept of glenohumeral instability.^14^,^16^ This condition bears two risk factors, i.e. engaging Hill–Sachs lesion and anterior glenoid deficiency, that have an additive effect of unrestricted posterior–anterior gliding of the humeral head without static anterior stabilization.

## MANAGEMENT OF PRIMARY STABILIZATION FAILURE

When the failure of primary anterior stabilization surgery is determined by functional scoring following estimation of the increased risk of re-dislocation after primary stabilization surgery, e.g. by the WOSI scoring, according to clinical examination of shoulder range of movements and stability, it should be expected that the pathophysiology of the remaining glenohumeral instability is identified based on the risk factors and predictive scoring (ISIS system). Subsequentially, further patient management planning is required in order to address specifically the cause of the initial surgery failure, with the foreseen expectation of the final successful shoulder stabilization.

Since shoulder instability, pain on exhort, and stiffness are of less functional importance in patients with functionally low demands, palliative non-surgical treatment following failure of primary shoulder stabilization might be of value if the severity of the disability due to the unstable shoulder can be controlled in part by pharmacological means and physiotherapy to strengthen the dynamic shoulder muscle stabilizers. Obviously, this solution cannot be applied to patients with functional demands, regardless of their age.

Therefore, in most clinical circumstances, revision shoulder stabilization surgery is necessary following failure of primary surgery. Revision surgery is primarily directed at resolving glenohumeral instability and pain, but the revision stabilization procedure could compromise the extent of shoulder range of movements as a secondary outcome; this should be discussed with the patient, especially in regard to his/her future physical abilities. A substantial arsenal of surgical solutions exists for different underlying causes of primary surgery failure, and revision surgery should be planned accordingly.

Anterior labral repair with or without capsular plication is highly effective for shoulder stabilization in patients with ligamentous laxity. When primary soft tissue repair fails due to suboptimal anchor placement, unbalanced capsule redundancy, humeral side ligamentous damage, and small non-engaging Hill–Sachs lesion of less than 20% anterior glenoid deficiency, the anterior labrum repair (Bankart repair) and capsule balancing by arthroscopic approach should suffice for functional outcome. Using this approach, shoulder stability with a good range of painless movement is expected to be achieved with revision surgery.

When the inferior glenoid deficiency is less than 20%, a bony procedure is not always considered,^3^ but when the bony loss exceeds 30% of the glenoid surface, the general opinion is that bone block implantation on anterior is advantageous, either by coracoid tip transfer (with the attached conjoint tendon providing an increase in glenoid surface, supplemented by suspension of the conjoint tendon, thereby providing dynamic stability to the head of humerus, e.g. Bristow technique,^17^ Latarjet technique),^18^ or autologous tricortical bone graft, which increases the glenoid surface.

The consideration for tricortical bone graft implantation is logical as an alternative technique for treating glenoid deficiency after a previous coracoid transfer that failed due to its malunion or non-union, but it is expected to have a less favorable functional outcome due to the high risk of postoperative shoulder stiffness.^19^

In rare cases when bone loss exceeds 45% of the glenoid surface, prosthetic replacement of the glenohumeral joint should be considered since treatment by bone grafting for extensive glenoid damage is not enough for mechanical glenohumeral stabilization.

A decision-making uncertainty exists if glenoid bone deficiency is in the intermediate range of 20%–30%. Under such circumstances, there is lack of substantial reported clinical information for the favored surgical treatment, either by the less extensive soft tissue procedure or by bone block implantation. In this “gray zone” of uncertainty, the relevant demographic data and personal risk factors (gender, age, contact sports activity, extent of ligamentous laxity) might help in making the surgical decision.

If the primary surgical stabilization failed because of significant bone loss in the humeral head and/or the glenoid, a more complicated surgical technique is required during revision surgery.

In the case of primary stabilization surgery failure due to large humeral head bony damage (Hill–Sachs lesion) that involves more than 20% of the surface, especially if the bony lesion is of an “engaging” type, autologous bone grafting with or without a “remplissage” procedure (filling the defect with infraspinatus tenodesis and posterior capsulodesis) is the favored surgical method, preferably using the arthroscopic approach that is expected to prevent glenohumeral dislocation by preventing the engaging mechanism of humeral impression on a glenoid rim.^20^ In the extreme situation of a combined engaging Hill–Sachs lesion and a large anterior glenoid deficiency with an “off-track” humeral gliding Hill–Sachs lesion, the surgical procedure should address the Hill–Sachs lesion fill and anterior block implantation onto the glenoid.

## SURGICAL OUTCOME

Failure rate of revision surgery following failed primary shoulder stabilization is below 20%,^3^ providing the failure definitions in both are similar, i.e. symptomatic shoulder instability, pain, and shoulder stiffness. We can assume with a high degree of certainty that the majority of revision failures are related to unsolved risk factors that led to the primary surgery failure, and partly to objective technical difficulties common in revision surgery due to the disturbed anatomical planes. But in spite of the expected higher technical challenge of revision surgery, surprisingly its failure rate is similar to primary stabilization surgery, i.e. at most 20%–25%. In general, the published data show that patients with glenohumeral dislocations are expected to suffer from final failure rate, after the sequence of primary and revision surgeries, at the maximal rate of 4%.

## Abbreviations

ISIS: Instability Shoulder Index Score
WOSI: Western Ontario Shoulder Instability Index
